# Disparities in the Concentrations of Essential/Toxic Elements in the Blood and Scalp Hair of Lymphoma Patients and Healthy Subjects

**DOI:** 10.1038/s41598-019-51973-5

**Published:** 2019-10-25

**Authors:** Muhammad Abdul Qayyum, Munir H. Shah

**Affiliations:** 1 Department of Chemistry, Division of Science & Technology, University of Education, Lahore, Pakistan; 2 Department of Chemistry, Quaid-i-Azam University, Islamabad, 45320 Pakistan

**Keywords:** Metals, Cancer

## Abstract

Lymphoma is one of the fastest growing malignancies worldwide and imbalance in the concentrations of trace elements can play a significant role in the onset and progression of the disease. Selected essential and toxic elements (Fe, Zn, Cu, Mn, Ni, Cr, Cd and Pb) were analysed in the blood & scalp hair of lymphoma patients (*n* = 59 & 58, respectively) and controls (*n* = 61 & 60, respectively) by atomic absorption spectrometry. On the average, Ni, Cr, Cu and Cd revealed significantly higher contents in the blood and scalp hair of the patients than the controls (*p* < 0.05). The correlation study showed significantly diverse relationships among the elements in blood & scalp hair of the two donor groups. Variations in the elemental levels with different types of lymphoma (non-Hodgkin and Hodgkin) were also evaluated. Disparities in the elemental concentrations were also investigated for various types of non-Hodgkin (diffuse large B-cell lymphoma, follicular lymphoma and peripheral T-cell lymphoma) and Hodgkin lymphoma (mixed cellularity, nodular lymphocyte predominant and nodular sclerosing), as well as for different stages (I, II, III & IV) of the cancer. Multivariate statistical analysis showed considerably divergent associations of the elements in the patients and controls. The study indicated profound alteration of the elemental levels in the patients; it may be implicated in elemental-induced disorders including lymphoma.

## Introduction

Manmade alteration of the environment such as food processing, water contamination, agricultural practices, fertilizers and various industrial emissions brought changes in the metals balance resulting in adverse health effects such as cancers^1^. Among cancers, lymphoma consists of a complex set of cancers of the haematopoietic system begins from lymph nodes, spleen, bone marrow, blood or other parts of the body and finally it forms a tumour^2^. Lymphoma is traditionally classified into two broad categories; Hodgkin lymphoma (HL) and non-Hodgkin’s lymphoma (NHL) and further subdivided into distinct entities based on characteristics, morphology, cytogenetic and molecular abnormalities^2,3^. The updated WHO classification recognized two groups of histological types of HL: nodular lymphocyte predominant HL and classic HL, the latter being further subdivided as, mixed cellularity HL, nodular sclerosis HL, lymphocyte depleted HL and lymphocyte-rich HL^3^. However, NHL encompasses a wide variety of disease subtypes namely follicular lymphoma, mantle cell lymphoma, marginal zone lymphoma, burkitt lymphoma, peripheral T-cell lymphoma, diffuse large B cell lymphoma etc. Staging of lymphoma is very important because it has different prognoses at each stage which is treated differently^2^. NHL is seen predominantly in developed/industrialized countries and is associated with age, gender, race, ultraviolet radiation, chronic antigenic stimulation or immunosuppression, hereditary factors, HIV/AIDS infection, Epstein Barr virus, hepatitis C virus, helicobacter pylori, hair dyes, alcohol, tobacco, occupational and environmental exposure of metals^4,5^. HL is mostly found in under developed countries and is frequently related to childhood social environment, Epstein-Barr virus, HIV infection, genetic factors, chemical and environmental factors such as exposure to metals, smoking and alcohol^6,7^.

Monitoring of trace and essential elements for human fluids and tissues has been of interest to researchers in the field of medical science and environmental chemistry. However, the choice of biological samples/matrices for determination is a difficult task. Numerous environmental monitoring programmes related to the distribution, absorption, kinetics and deleterious effects of toxic elements in biological systems have been carried out^8^. Blood, breast milk, urine, foetal membranes, hair, placenta, saliva and nails are often used as complementary diagnostic tissue for measuring trace elements and each tissue provides unique clinical information about elemental levels of exposed individuals^9,10^. Furthermore, analyses of blood and hair have been linked with future development of diseases thus manifesting their potential as predictive and preventive markers in addition to monitor the current status and symptoms of elemental imbalances. Blood is the easiest and most commonly used matrix for assessing the status of trace elements. However, blood concentrations may be affected by other factors making interpretation rather difficult. Moreover, blood indicates the amount of an agent actually present in the body and reflects xenobiotic exposure based on a very short or limited period, i.e. hours or days, whereas, hair display this exposure, after weeks or months due to the structure notably keratin, a group of proteins containing disulphide bonds^9,10^. The concept behind hair analysis offers benefits revolving around its relatively fast growth, behaviour and ease of non-expensive, non-invasive collection, transportation, easy manipulation, high stability at room temperature and relatively higher elemental concentration than other specimens^10^. Depending on the specific elements and the time after exposure and other pathological conditions such as haematological or kidney disease, blood may not be good indicators of body burdens^11^. Some limitations associated with the use of hair include the differential washing procedures, effects of cosmetic treatment, dying, lack of standardization, bleaching, permanent waving the differentiation between endogenous (xenobiotics reach the hair matrix through blood) and exogenous (atmospheric deposition) analyte and sampling variations at different sites^8^. On comparative basis, blood analysis reflected short-term exposure while hair analysis revealed long-term exposure to the pollutants/nutrients.

Several epidemiological studies on trace and essential elements in lymphoma have been published in the developing countries but most of these studies are focused on one or two elements^12–14^. Therefore, there is dire need to evaluate relatively greater number of essential/toxic elements to explore their mutual variations in relation to the progression of the disease. In addition, limited data are available in published literature from Pakistan in relation to the levels of trace and essential elements for the lymphoma patients^15^. Major aim of this study is to find out any relationship between the lymphoma and concentrations of selected essential/toxic trace elements. Therefore, main objectives of the present study are; 1) to evaluate the levels of essential and toxic trace elements (Fe, Zn, Cu, Mn, Ni, Cr, Cd and Pb) in the blood and scalp hair of lymphoma patients and controls with matching age, habitat and eating habits; 2) to find out any mutual variations between the elemental concentrations by correlation study; 3) to explore the multivariate methods for the apportionment of toxic and essential elements in the blood and scalp hair of the patients and controls; 4) to assess the levels of elements with respect to the types, subtypes and stages, thereby investigating whether these elements had any presumptive link in the diagnosis and/or prognosis of lymphoma in patients.

## Materials and Methods

### Study population

The scalp hair and blood samples were collected from the newly diagnosed lymphoma patients hospitalized at Nuclear Oncology & Radiotherapy Institute (NORI), Islamabad, Pakistan. All subjects were selected on volunteer basis. The study was reviewed and approved by the ethical review committee of NORI, Islamabad (App. No. QAUC/2013/NORI-A98) and conducted according to the Helsinki Declaration. Written informed consent was obtained from all participants before collecting the sample. These lymphoma patients, with ages ranging from 24 to 86 years, were recruited into the study at the onset of disease, and before undergoing any treatment such as surgery, chemotherapy or radiotherapy. Neither of the subject groups had taken any mineral supplement and/or vitamin for at least six mounts before the study. All lymphoma patients were biopsy proven diagnosis and types, subtypes and stages were identified by histopathological, radiological and clinical examinations. The healthy subjects (with ages ranging from 24 to 65 years) were also recruited on volunteer basis from the same localities with matched age groups, similar socio-demographics and food habits. The subjects were initially briefed about the purpose and objectives of the study and and then a written consent was obtained. At the start of the study, information about demographic characteristics including age, gender, dietary intake, smoking habits, type of cancer, duration, cancer stage, medication use, occupational, residential and family histories etc., were also obtained. None of the patients or controls was consuming alcohol on continuous basis. Morphometric parameters including height, body weight, blood pressure and biochemical data were assessed in the institute before sample collection.

### Collection and processing of the blood samples

Approximately 3 mL venous blood was withdrawn from an antecubital vein using standard phlebotomy technique from each participant. Blood samples were collected in separate metal free vacutainer tubes (BD Vacutainer Ref. 366,430) with out anticoagulants at room temperature. The samples were stored in refrigerator until further biochemical determinations. For digestion, whole blood sample (about 5 mL) was taken into the digestion flask and digested with HNO_3_–HClO_4_ (10:1 v/v) mixture to eliminate interference from the organic matrix. The mixture was gently heated on a hot plate until the brown fumes given off by the reaction turned white. The digestion flask was brought down from the hot plate to cool to room temperature. The concentrated mixture (containing the sample) was diluted to proper volume with doubly distilled water^9^. Blanks (without blood sample) were also prepared following complete procedure with each batch of the 5 samples. The completion of digestion procedure was confirmed by physical examination and emergence of white dense fumes as already reported in literature^9–11^.

### Collection and processing of the hair samples

Scalp hair samples were collected from occipital region in a quantity of about 3 to 5 g. Collection was performed using a pair of plastic scissors to prevent external contamination of the samples. The samples were packed into polyethene bags together with the filled questionnaire for each individual, duly labelled with relevant codes. Washing/cleaning procedure prior to analysis is significant to remove exogenous contaminants which include the environment (dust, air, water, oil, grease) and hair treatments (dyes, perms). Therefore, scalp hair washing procedure was carried out to provide an accurate assessment of endogenous metal contents^8^. The samples were cut into approximately 2–3 cm pieces long and mixed to allow a representative sub-sampling of the hair specimen. After cutting each sample was thoroughly mixed and soaked in detergent solution (50 mL) in a flask and shaken on an auto-shaker for 30 minutes at 320 vibrations per minute and were left undisturbed for 2 h. Each sample was washed with plentiful water to remove possible detergent. Then 30 mL Triton X-100 (0.5% v/v) was added and again shaken on an auto-shaker for 20 minutes. Next, samples were rinsed with an abundant amount of doubly distilled water and allowed to drain. After the washing process, the samples were dried in an electric oven at 70 °C for overnight and were left to cool at room temperature in a desiccator containing silica gel as the desiccant and finally weighed by an analytical balance^10^. Then a weighed portion of clean and dried scalp hair sample (~2 g) was introduced in a conical flask to which 10 mL of concentrated HNO_3_ (65%) was added and the mixture was heated on a hot plate at about 80 °C for 10 minutes. After cooling, 5.0 mL of HClO_4_ (70%) was added to the solution, with subsequent heating to a soft boil until transparent solution was obtained. After digestion, the solutions were properly diluted by the addition of distilled water^16^. Blanks were routinely prepared following the same digestion process but without the hair sample.

### Quantification of the elements

Selected trace elements (Fe, Zn, Cu, Mn, Ni, Cr, Cd and Pb) in the samples were determined using a flame atomic absorption spectrophotometer (Shimadzu AA-670, Japan) under optimized operational conditions as shown in Table 1. All the reagents were of analytical grade and for the preparation of standards and samples, doubly distilled water was used. Stock solution containing 1000 mg/L of each element was used to prepare the fresh working standards just before the analysis on the instrument. All the measurements were performed in triplicate and run separately onto the spectrophotometer to pool the average concentrations. For accuracy of the methodology, standard reference materials (Animal Serum, NIST SRM 1598a, Human Hair, GBW 07601) were used, which showed very good recoveries (97–101%) as shown in Table 1. The samples were also analyzed at an independent laboratory for comparison of the results and a maximum of ±2.5% difference was observed in the results of two laboratories. Generally, the contribution of the blank was <5% of the measured concentrations in the samples.

**Table 1:** Optimum analytical conditions for the elemental analyses along with their limits of detection & quantification and certified versus measured concentrations of selected elements in standard reference materials.

		Fe	Zn	Cu	Mn	Ni	Cr	Cd	Pb
Wavelength (nm)		248.3	213.9	324.8	279.5	232	357.9	228.8	217
Slit width (nm)		0.2	0.5	0.5	0.4	0.15	0.5	0.3	0.3
Lamp current (mA)		8	4	3	5	4	5	4	7
Acetylene flow rate (L/min.)		2	2	1.8	1.9	1.7	2.6	1.8	1.8
Limit of Detection (µg/L)		6	2	4	3	6	6	4	11
Limit of Quantification (µg/L)		18	6	13	10	19	18	13	29
NIST SRM 1598a	Certified Level*	1680	880	1580	1.78	0.94	0.33	0.048	—
	Measured Level*	1651	892	1560	1.73	0.95	0.32	0.046	—
	Recovery (%)	98	101	99	97	101	97	96	—
GBW 07601	Certified Level**	54	190	10.6	6.3	0.83	0.37	0.11	8.8
	Measured Level**	54	188	10.8	6.2	0.85	0.36	0.11	8.6
	Recovery (%)	100	99	102	98	102	97	98	98

*µg/L; **µg/g.

### Statistical analysis

The quantified results were subjected to univariate and multivariate analysis to classify the relationship among the measured variables. STATISTCA software was used for the statistical computation of the results. General descriptive statistics (range, mean, standard error (SE) and skewness) was employed to present the main findings of this study. Student *t*-test was used to verify whether there were significant differences between the elemental levels among lymphoma patients and controls. The *p* < 0.05 was set at statistical significance. Spearman correlation was used to verify the inter-element correlations in blood and scalp hair. The multivariate PCA and CA were computed to understand the complex phenomenon of the associations and apportionment among the elemental distribution in blood and scalp hair samples of patients and controls. The PCA was done by applying varimax normalized rotation on the data-set, and the CA was applied to the standardized matrix of samples. The Ward’s method is used and the results of CA are displayed in the form of dendrogram which depicts the levels of similarity between different variables^17^. PCA is mainly used as a method of data reduction, and simplification of the original data matrix, which is believed to have specific components meaning and preserving the maximum variability contained in the data. Further, it finds the relations in data of high dimension simultaneously^18^. CA is an unsupervised multivariate statistical method; it recognizes the groups of objects or variables based on their similarity. The greater is the similarity, the smaller is the distance. Thus, CA builds a predictive model that allows to plug in the numbers of new cases and to predict mutual properties based on an overall group membership^16^.

## Results and Discussion

### Demographic characteristics of the donors

The demographic characteristics related to the lymphoma patients (hereafter called ‘patients’) and matched healthy donors (hereafter called ‘controls’) are summarized in Table 2. Majority of them in both groups were vegetarians. Almost half of the patients belonged to rural areas and most of the patients were not using tobacco on continuous basis in contrast to controls. The relative proportion of the healthy donors was more or less similar as those of the patients. Patients included in the present study were commonly suffering from NHL (52–53%) and HL (47–48%). The NHL patients were further divided into histological subtypes; diffuse large B-cell lymphoma (39–41%), peripheral T-cell lymphoma (31–32%) and follicular lymphoma (28–29%). Likewise, HL patients encompassed further into mixed cellularity HL (38–39%), nodular sclerosing HL (32–34%) and nodular lymphocyte predominant HL (28–29%). Regarding the histopathological staging, the highest number of cases was found at stage I (29%) followed by 26–27% at stage II, 25% at stage IV and 19–20% at stage III as shown in Table 2.

**Table 2:** Characteristics of the subjects.

Characteristics	Blood		Scalp Hair	
	Patients	Controls	Patients	Controls
*n*	59	58	61	60
*Age (years)*				
Range	24–86	25–65	24–86	24–60
Mean	57.19	43.93	57.05	42.24
*Gender*				
Female	22 (37%)	18 (31%)	23 (38%)	19 (32%)
Male	37 (63%)	40 (69%)	38 (62%)	41 (68%)
*Diet*				
Vegetarian	33 (56%)	37 (64%)	34 (56%)	39 (65%)
Non-vegetarian	26 (44%)	21 (36%)	27 (44%)	21 (35%)
*Habitat*				
Urban	27 (46%)	25 (43%)	28 (46%)	25 (42%)
Rural	32 (54%)	33 (57%)	33 (54%)	35 (58%)
*Tobacco Use (Smoking)*				
No use	35 (59%)	36 (62%)	37 (61%)	38 (63%)
Use	24 (41%)	22 (38%)	24 (39%)	22 (37%)
*Types (histology) of lymphoma*				
Non-Hodgkin lymphoma	31 (53%)		32 (52%)	
Hodgkin lymphoma	28 (47%)		29 (48%)	
*Types of non-Hodgkin lymphoma*				
Diffuse large B-cell lymphoma	12 (39%)	—	13 (41%)	—
Follicular lymphoma	09 (29%)	—	09 (28%)	—
Peripheral T-cell lymphoma	10 (32%)	—	10 (31%)	—
*Types of Hodgkin lymphoma*				
Nodular lymphocyte predominant HL	08 (29%)		08 (28%)	
Mixed cellularity HL	11 (39%)	—	11 (38%)	—
Nodular sclerosing HL	09 (32%)	—	10 (34%)	—
*Stages of lymphoma*				
Stage I	17 (29%)	—	18 (29%)	—
Stage II	16 (27%)	—	16 (26%)	—
Stage III	11 (19%)	—	12 (20%)	—
Stage IV	15 (25%)	—	15 (25%)	—

### Distribution of the elements in blood

Basic statistical parameters for the distribution of the trace and essential elements in blood samples of patients and controls are represented in Table 3. In blood samples of the patients, average concentrations revealed dominant contribution for Fe (42887 µg/dL), followed by Zn (614.9 µg/dL), Ni (257.8 µg/dL) and Cu (108.5 µg/dL). However, relatively lower mean concentrations were found for Cr (59.43 µg/dL), Pb (52.05 µg/dL), Cd (50.03 µg/dL) and Mn (15.92 µg/dL). On the mean basis, the decreasing trend of elemental concentrations in the patients revealed following order: Fe > Zn > Ni > Cu > Cr > Pb > Cd > Mn. Among the selected elements, Fe and Zn exhibited relatively higher dispersion as revealed by higher SE value, while Mn, Cd, Pb, Cu and Cr showed relatively lower dispersion manifested by lower values of SE. Large skewness values for Mn and Ni supported asymmetrical distribution of the elements, while, all other elements exhibited almost symmetrical distribution in the blood of the patients as shown by lower skewness values.

**Table 3:** Statistical distribution parameters for selected elemental concentrations in the blood (µg/dL) and scalp hair (µg/g) of the lymphoma patients and controls.

		Patients					Controls					*p*-value
		Min	Max	Mean	SE	Skew	Min	Max	Mean	SE	Skew	
Blood (µg/dL)	Fe	14425	56745	42887	1025	−0.834	13368	49057	38579	793.9	−1.189	NS^a^
	Zn	119.8	1025	614.9	27.68	−0.116	430.1	1259	727.6	22.32	0.824	NS^a^
	Cu	11.60	178.8	108.5	5.145	−0.484	13.02	168.4	79.08	3.929	0.480	<0.05
	Mn	0.943	66.32	15.92	1.561	1.742	4.906	107.6	26.29	2.436	1.930	<0.05
	Ni	93.68	605.1	257.8	14.88	1.017	4.245	511.0	188.4	17.21	0.666	<0.05
	Cr	1.887	143.1	59.43	5.070	0.231	1.038	130.1	34.95	4.927	1.230	<0.05
	Cd	7.830	124.2	50.03	3.759	0.846	3.113	64.91	25.04	2.002	0.910	<0.05
	Pb	1.132	136.0	52.05	4.645	0.857	4.151	187.3	77.09	6.192	0.415	<0.05
Scalp Hair (µg/g)	Fe	3.750	28.60	15.05	0.754	0.535	6.850	35.70	19.13	0.834	0.425	NS^a^
	Zn	91.15	369.1	205.6	6.431	0.364	96.90	481.4	247.3	9.313	1.275	NS^a^
	Cu	6.500	18.25	10.99	0.318	0.922	5.550	15.50	9.962	0.259	0.486	NS^a^
	Mn	0.150	10.73	2.038	0.220	3.030	0.400	13.73	3.262	0.303	2.280	<0.05
	Ni	0.300	20.35	9.314	0.684	0.348	0.050	16.90	5.836	0.589	0.697	<0.05
	Cr	0.300	5.750	3.013	0.163	−0.380	0.100	5.600	1.732	0.199	1.075	<0.05
	Cd	0.200	1.800	0.615	0.045	1.068	0.050	0.850	0.305	0.025	1.067	<0.05
	Pb	1.100	13.60	5.160	0.458	1.216	1.035	14.200	7.077	0.424	0.396	<0.05

^a^NS-non significant.

In the case of controls, markedly higher mean level was found for Fe (38579 µg/dL), followed by relatively lower concentrations of Zn (727.6 µg/dL), Ni (188.4 µg/dL), Cu (79.08 µg/dL) and Pb (77.09 µg/dL). Lowest mean concentrations were noted for Cr (34.95 µg/dL), Mn (26.29 µg/dL) and Cd (25.04 µg/dL). The observed decreasing trend of mean elemental levels in the controls exhibited following order; Fe > Zn > Ni > Cu > Pb > Cr > Mn > Cd. Relatively larger dispersion in terms of SE was pointed out by Fe, however, a noticeably higher asymmetry was noted for Mn, Cr and Fe in the blood of controls.

**Figure 1:**
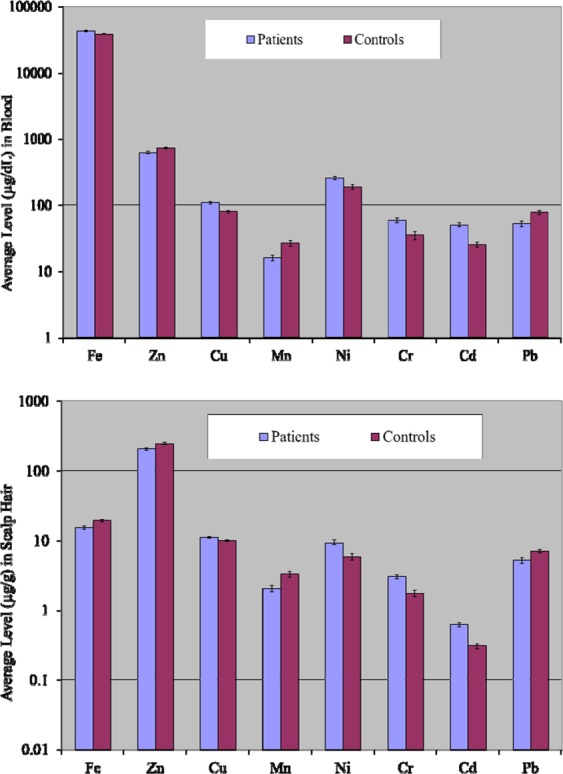
Comparative average concentrations of selected elements (±SE) in the blood and scalp hair of lymphoma patients and controls.

Two-tailed Student’s *t*-test assuming unequal variance (*p* < 0.05) of the data illustrated that there were lack of significant variations between the concentrations of Fe and Zn in the blood of the patients and controls, whereas average concentrations of Cd, Cr, Cu and Ni were significantly elevated and mean Pb and Mn levels were significantly lower (*p* < 0.05) in the blood of the patients compared to the healthy donors. In addition, Zn level was found to be considerably higher in the blood of controls compared with the patients (Fig. 1).

### Distribution of the elements in scalp hair

The concentrations of selected elements in the scalp hair of patients and controls as shown by basic statistical parameters are given in Table 3. On the average, elevated levels were found for Zn (205.6 µg/g), followed by relatively lower concentrations of Fe (15.05 µg/g), Cu (10.99 µg/g), Ni (9.314 µg/g) and Pb (5.160 µg/g), while Mn and Cd were estimated at the lowest levels in the scalp hair of the patients. Overall, the elemental contents in the patients revealed following decreasing order; Zn > Fe > Cu > Ni > Pb > Cr > Mn > Cd. Large skewness values for Mn, Cd and Pb exhibited their asymmetric distribution in the scalp hair of the patients whereas Zn exhibited relatively higher dispersion as manifested by high SE value.

In the case of controls, on the mean scale, predominantly higher concentration was noted for Zn (247.3 µg/g), followed by, Fe (19.13 µg/g), Cu (9.962 µg/g), Pb (7.077 µg/g) and Ni (5.836 µg/g). Lowest average levels were shown by Cr (1.732 µg/g) and Cd (0.305 µg/g). The average concentrations of the elements in the controls revealed following order: Zn > Fe > Cu > Pb > Ni > Mn > Cr > Cd. Mean concentrations of Cd, Cr, Cu and Mn exhibited relatively normal distribution pattern in the controls as shown by their SE values. Large skewness and kurtosis values for Mn, Zn, Cr and Cd showed asymmetrical distribution of these elements in the scalp hair of the controls.

Comparison of the elemental levels by student’s *t*-test in the scalp hair of the patients and controls revealed almost similar levels and hence insignificant variations for Cu, Fe and Zn. However, average concentrations of Cd, Cr and Ni were significantly higher (*p* < 0.05) in the patients and Pb was significantly higher in the controls (*p* < 0.05) as shown in Fig. 1. Fe and Zn levels were considerably higher in the controls when compared to those of the patients, thus manifesting the deficiency of these essential nutrients in the patients.

### Correlation study

The interest in correlations may assist in the interpretation of the meaningful measurements. Such an approach should also help to identify common factors inducing the observed elemental relationships^19^. A high value of the coefficient reveals high linear correlation between the contents of two elements. Table 4 shows the Spearman correlation coefficient (*r*) matrix between the elements in the blood and scalp hair of the lymphoma patients and controls. Most of the concentrations were correlated positively; however, only few of them were correlated negatively. No significant correlation was observed between the elements which manifested their independent variations in the blood of the patients. The counterpart data for the controls (Table 4) exhibited significant relationship between Zn–Fe (*r* = 0.328), Cr–Fe (*r* = 0.318) and Ni–Fe (*r* = 0.314). A few negative correlations were also observed but they were not significant.

**Table 4:** Correlation coefficient (*r*)^a^ matrix of selected elements in the blood and scalp hair of lymphoma patients (below the diagonal) and controls (above the diagonal).

		Fe	Zn	Cu	Mn	Ni	Cr	Cd	Pb
Blood	Fe	1	**0.328**	0.270	0.117	**0.314**	**0.318**	0.149	−0.101
	Zn	0.205	1	0.040	0.004	0.151	0.062	0.155	0.109
	Cu	0.035	−0.115	1	−0.123	−0.173	0.283	−0.046	−0.046
	Mn	0.069	0.177	−0.054	1	0.044	0.004	0.176	−0.054
	Ni	−0.150	−0.186	0.136	−0.070	1	−0.003	−0.006	0.077
	Cr	−0.151	−0.027	0.084	−0.037	0.081	1	0.097	−0.085
	Cd	0.213	0.307	−0.019	0.196	−0.189	0.023	1	−0.188
	Pb	0.046	0.020	0.038	−0.034	0.072	−0.177	0.198	1
Scalp Hair	Fe	1	0.028	0.138	−0.100	−0.214	**0.362**	**0.320**	0.138
	Zn	−0.172	1	−0.042	−0.043	0.177	0.001	−0.145	**0.347**
	Cu	0.263	0.100	1	**0.371**	−0.152	0.211	0.096	−0.070
	Mn	−0.007	−0.057	−0.132	1	−0.243	0.101	−0.005	−0.103
	Ni	0.032	−0.036	0.041	−0.140	1	−0.012	0.145	−0.152
	Cr	0.001	0.013	0.037	0.028	−0.014	1	**0.356**	0.069
	Cd	−0.033	0.022	−0.179	0.026	0.011	−0.053	1	−0.129
	Pb	−0.115	0.207	0.135	0.189	0.166	0.045	−0.043	1

^a^*r*-values > 0.311 or <– 0.311 are significant at *p* < 0.001.

In the case of scalp hair of the patients, no significant positive or negative correlation was observed between the elements as shown in Table 4. For controls, significant positive correlations were noted between Mn–Cu (*r* = 0.371), Cr–Fe (*r* = 0.362), Cd–Cr (*r* = 0.356), Pb–Zn (*r* = 0.357) and Cd–Fe (*r* = 0.320). Overall, the correlation behaviour of the elements in controls remained noticeably diverse compared to the lymphoma patients, which may be attributed to the disproportions of the essential and toxic elements in the patients.

### Multivariate analysis

**Figure 2:**
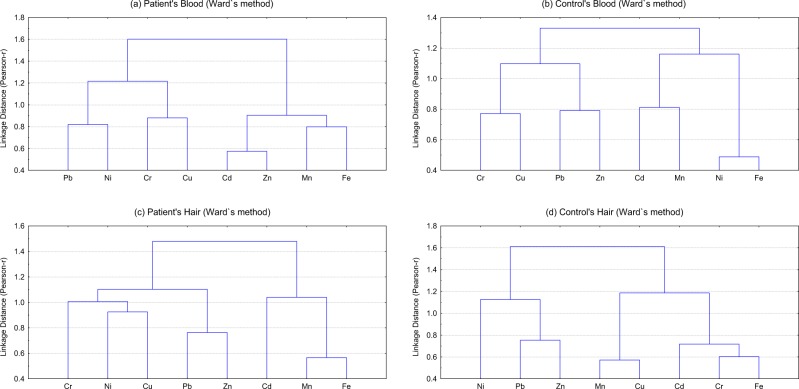
Cluster analysis of selected elements in the blood and scalp hair of lymphoma patients and controls.

The principal component (PC) loadings of the elements in blood of the patients and controls are presented in Table 5. The corresponding CA based on Ward’s method of selected elements is portrayed in Fig. 2. For blood of the patients, PC 1 revealed dominant loadings for Mn, Fe, Cd and Zn, duly supported by strong clusters between Mn & Fe and Cd & Zn in the CA. Loading of these elements may be related to the environmental pollution coupled with the dietary life style of the patients. Study of Jomova and Volko^20^ advocated the role of Cd and Fe in the enhancement of oxidative stress which is ultimately responsible for the development of haematological cancer including lymphoma. It is believed that Cd is a significant toxic and carcinogenic element because it may act as a catalyst to oxidation reactions resulting in the generation of free radicals that damage the tissue of the body^20^. On the other side, Zn is an antioxidant, has anti-inflammatory actions and may decrease oxidative stress^21^. Moreover, Cd enhanced the levels of lipid peroxides and alters the activity of several antioxidant enzymes including Zn-SOD in rats^20^. PC 2 observed maximum loadings for Ni and Pb and a mutual cluster for Ni and Pb is also shown by CA, most probably originating from the anthropogenic contributions (automobile emissions). Similarly, significant loadings for Cr and Cu noted in the third PC 3 with a joint cluster (Cr & Cu) in CA. These metals mostly affiliated by food habits & environmental pollution^10^.

**Table 5:** Principal component loadings of selected elements in the blood and scalp hair of the lymphoma patients and controls.

	Blood						Scalp hair							
	Patients			Controls			Patients				Controls			
	PC 1	PC 2	PC 3	PC 1	PC 2	PC 3	PC 1	PC 2	PC 3	PC 4	PC 1	PC 2	PC 3	PC 4
Eigen value	1.894	1.257	1.139	1.869	1.506	1.26	1.682	1.347	1.182	1.006	2.033	1.522	1.3	1.016
Total Variance (%)	23.68	15.72	14.24	23.36	18.83	15.75	21.03	16.84	14.77	12.58	25.41	19.03	16.25	12.7
Cumulative Variance (%)	23.68	39.4	53.63	23.36	42.19	57.94	21.03	37.86	52.63	65.22	25.41	44.44	60.69	73.39
Fe	0.514	−0.186	0.047	0.807	0.162	0.357	0.835	−0.078	0.215	0.071	0.798	0.080	0.218	0.048
Zn	0.543	−0.329	−0.381	0.455	−0.387	0.319	−0.32	0.715	0.164	−0.002	−0.029	0.041	−0.163	0.903
Cu	−0.086	0.229	0.626	−0.057	−0.187	0.755	0.259	0.147	0.757	0.194	−0.481	0.687	−0.054	−0.027
Mn	0.714	0.072	0.257	0.125	0.495	−0.167	0.773	−0.036	−0.168	−0.108	0.112	0.917	−0.021	0.142
Ni	−0.148	0.553	0.044	0.843	0.056	−0.253	0.070	−0.212	−0.133	0.834	0.026	−0.121	0.890	−0.170
Cr	0.155	−0.378	0.738	0.146	0.228	0.729	0.175	0.634	−0.321	−0.046	0.710	−0.246	0.042	0.127
Cd	0.774	0.052	−0.227	0.158	0.685	0.152	−0.199	−0.220	0.665	−0.191	0.686	0.061	−0.352	−0.289
Pb	0.075	0.841	−0.016	−0.191	0.732	0.064	−0.196	0.368	0.241	0.620	0.130	0.221	0.538	0.546

The CA of the element data pertaining to the controls blood samples in the form of dendrogram (Fig. 2) revealed very strong clusters of Ni–Fe, Cd–Mn, Cu–Cr and Pb–Zn. PC 1 with highest variance of the data revealed maximum loadings for Fe and Ni. These elements can be traced to originate as environmental pollutants and nutritional sources. PC 2 manifested elevated loadings for toxic elements Cd, Mn and Pb for controls. These elements were most probably originating from environmental pollution particularly automobile emissions and fuel combustion. The CA results verified the PCA findings. The last PC for the controls showed dominant loadings of Cu and Cr with a similar cluster in CA; these elements are mainly regulated by internal body metabolism and environmental contamination. This multivariate method thus advocated different elemental distribution and apportionment pattern in the blood of lymphoma patients compared with the controls.

The PC loadings of the elements in the scalp hair of the patients and controls extracted by using varimax-normalized rotation on the data are also displayed in Table 5. Four PCs were extracted with eigen values greater than 1 exhibiting more than 65% of the cumulative variance of the patient’s element data. Based on Ward’s method, the corresponding CA is shown in Fig. 2, which revealed four clusters: (1) Fe-Mn-Cd, (2) Zn-Pb, (3) and Cu-Ni-Cr. PC 1 represented by Fe and Mn dully supported by CA, which were originated mostly from the food habits. PC 2 evidenced highest loadings for Zn and Cr, while Cd and Cu showed maximum loadings for PC 3, which indicated altered body metabolism that may be attributed to the onset and progress of the disease. Last PC constituted dominant loadings for Pb and Ni in the scalp hair of the patients. This PC comprising of carcinogenic metals exhibited the contribution from anthropogenic activities (automobile emissions, burning fuels, mining and metal processing) to the donors^22^.

In case of controls, PC 1 demonstrated highest loadings for Fe, Cr and Cd, with strong clusters of Fe–Cr–Cd as observed in CA (Fig. 2), which may be traced to originate mostly from the food habits and environmental contaminants. PC 2 revealed higher contributions of Mn and Cu, duly supported by a strong cluster in CA. Likewise PC 3 for the scalp hair of controls observed dominant loadings for Ni. Elevated loadings for Zn and Pb were indicated in PC 4 with considerable loadings of Pb, Zn, and Ni in CA. Loadings of these metals were mostly contributed by anthropogenic pollutants and food habits of the donors. The present results unfold that, in the case of the patients, the metabolism of essential elements was significantly altered and/or affected by the toxic trace elements^10,16^.

### Comparison among various types of lymphoma

**Figure 3:**
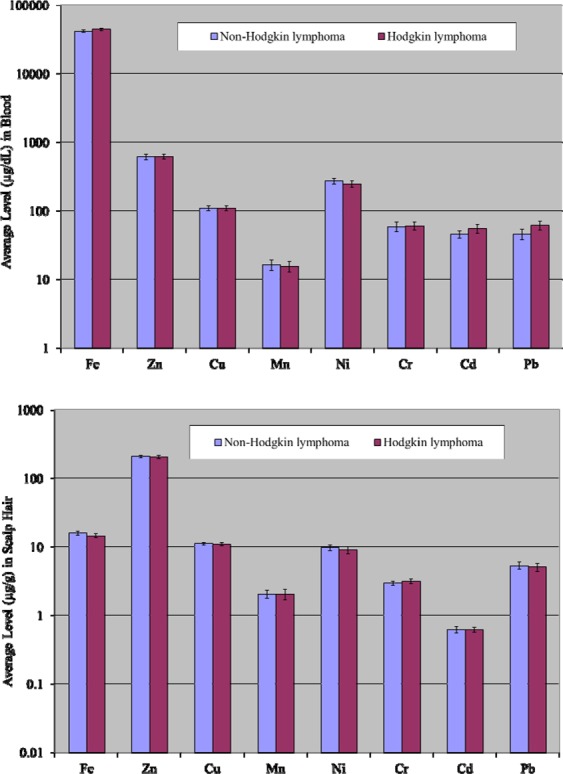
Comparative average concentrations of selected elements (±SE) in the blood and scalp hair of patients based on lymphoma types.

Comparative evaluation of mean elemental concentrations in the blood of various types of lymphoma patients (i.e., non-Hodgkin lymphoma and Hodgkin lymphoma) are shown in Fig. 3. In case of non-Hodgkin lymphoma patients, Mn and Ni displayed comparatively higher concentrations in the blood, while average levels of Fe, Cd and Pb were appreciably elevated in Hodgkin lymphoma patients’ blood. Mean levels of Cu, Cr and Zn were not appreciably different in the blood of non-Hodgkin and Hodgkin lymphoma patients.

In the case of scalp hair, average concentrations of Fe, Ni and Pb exhibited elevated contributions in non-Hodgkin lymphoma, while mean concentration of Cr was noted to be in excess in Hodgkin lymphoma patients as shown in Fig. 3. Nevertheless, Mn, Zn, Cu and Cd exhibited almost equivalent average concentrations in the scalp hair of non-Hodgkin and Hodgkin lymphoma patients.

### Comparison among various types of non-Hodgkin lymphoma

**Figure 4:**
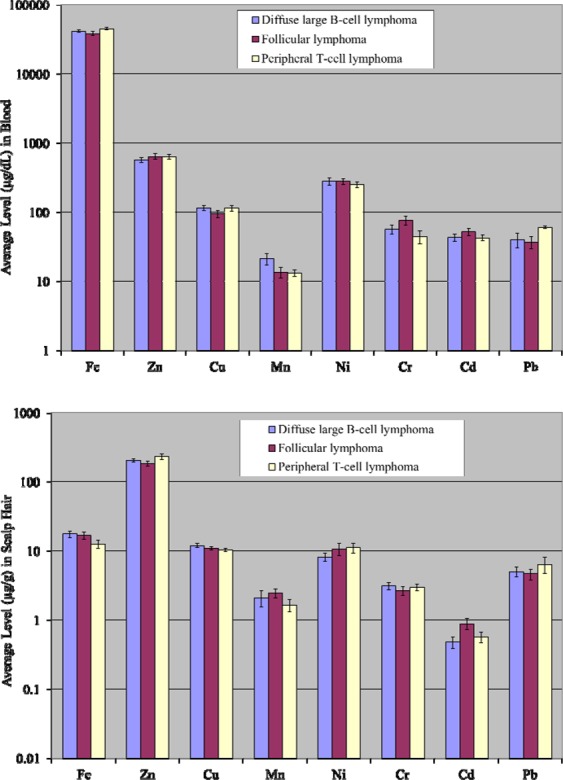
Comparative average concentrations of selected elements (±SE) in the blood and scalp hair of patients based on non-Hodgkin lymphoma types.

Comparative appraisal of trace and essential elements in the blood of various types of the non-Hodgkin lymphoma patients (diffuse large B-cell lymphoma, follicular lymphoma and peripheral T-cell lymphoma) are shown in Fig. 4. Highest concentration of Mn was found in the blood of diffuse large B-cell lymphoma patients, while average levels of Cr and Cd were relatively higher in the blood of follicular lymphoma patients. Furthermore, average levels of Fe and Pb were observed to be exceedingly higher in the blood of peripheral T-cell lymphoma patients. Some of the elements (Fe, Ni and Pb) exhibited almost equivalent mean concentrations in diffuse large B-cell lymphoma patients and follicular lymphoma patients, while, mean concentrations of Zn and Mn were not dissimilar in the blood of follicular lymphoma patients and peripheral T-cell lymphoma patients.

Mean concentrations of the elements in the scalp hair of different types of non-Hodgkin lymphoma patients are represented in Fig. 4. The mean contents of Fe and Cu were higher in the scalp hair of diffuse large B-cell lymphoma patients, while average concentrations of Cd and Mn were observed to be elevated in the scalp hair of follicular lymphoma patients. Similarly, average levels of Zn, Ni and Pb were relatively higher in the scalp hair of peripheral T-cell lymphoma patients.

### Comparison among various types of Hodgkin lymphoma

**Figure 5:**
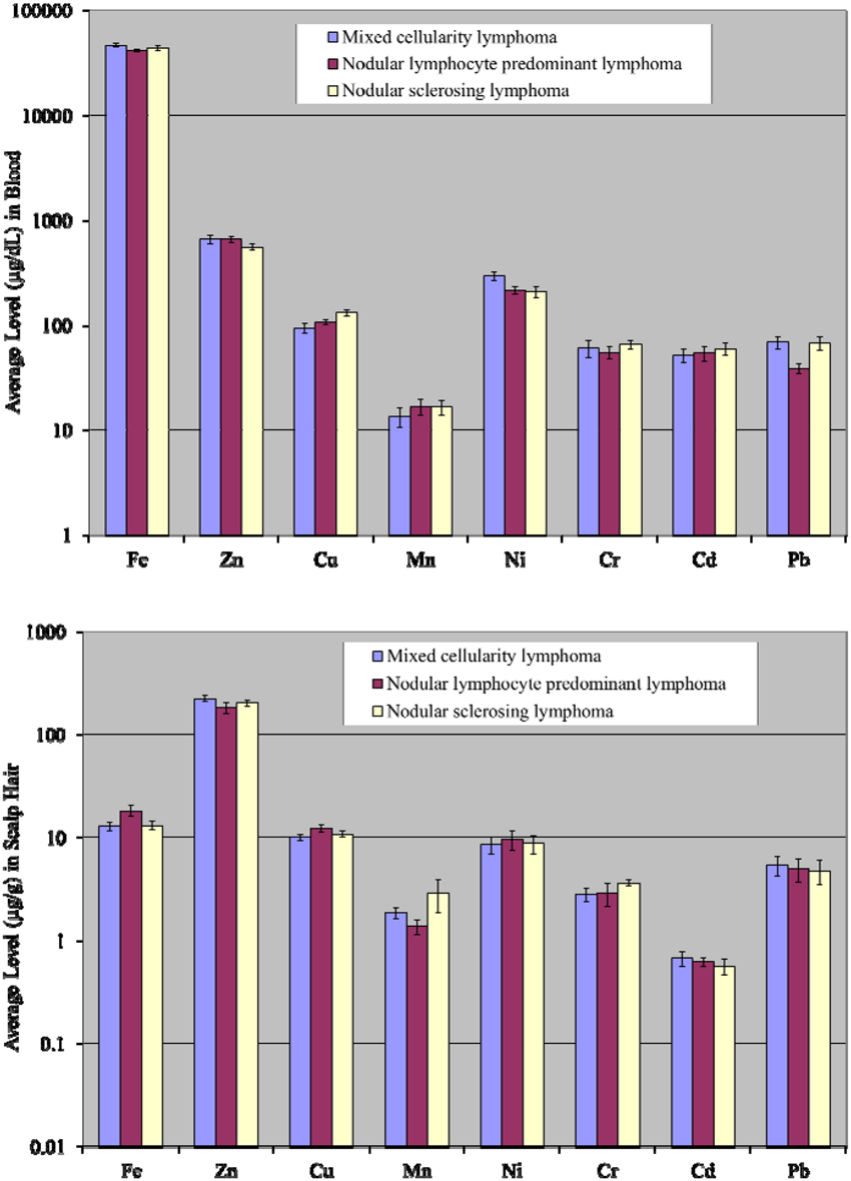
Comparative average concentrations of selected elements (±SE) in the blood and scalp hair of patients based on Hodgkin lymphoma types.

Comparison of the elemental concentrations in the blood of various types of Hodgkin lymphoma patients (i.e., mixed cellularity lymphoma, nodular lymphocyte predominant lymphoma and nodular sclerosing lymphoma) is given in Fig. 5. Average level of Ni was significantly higher in mixed cellularity lymphoma patients, whereas, Cu, Cr and Cd revealed higher levels in nodular sclerosing lymphoma patients. However, average concentrations of Mn and Zn were comparable in nodular lymphocyte predominant lymphoma patients and nodular sclerosing lymphoma patients, whereas Zn and Cd showed similar mean values in mixed cellularity lymphoma and nodular lymphocyte predominant lymphoma patients. Conversely, some of the elements (Fe, Cr and Pb) exhibited lowest concentrations in the blood of nodular lymphocyte predominant lymphoma patients.

A comparison of average elemental levels in the scalp hair of different types of Hodgkin lymphoma patients is shown in Fig. 5. Mean concentrations of Cd, Zn and Pb were noted to be in excess in the scalp hair of mixed cellularity lymphoma, whereas mean concentrations of Fe, Cu and Ni were relatively higher in nodular lymphocyte predominant lymphoma patients. Moreover, Mn and Cr levels were found to relatively higher in nodular sclerosing lymphoma patients.

### Comparison based on stages of lymphoma

**Figure 6:**
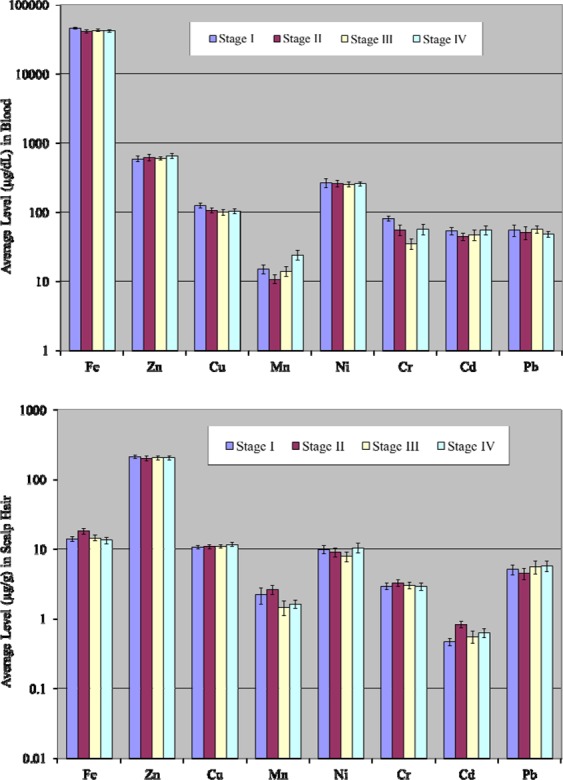
Comparative average concentrations of selected elements (±SE) in the blood and scalp hair of patients based on lymphoma stages.

Comparative average elemental concentrations in the blood and scalp hair of the patients at different stages of lymphoma are depicted in the Fig. 6. The average levels of Cu and Cr were noticeably higher at stage I, Pb level was higher at stage III and Mn was elevated at stage IV in the blood of the patients. Mean levels of Mn and Cd followed the decreasing order; Stage IV > Stage I > Stage III > Stage II.

In the case of scalp hair, mean concentrations of Fe, Mn, Cr and Cd were found to be noticeably higher at stage II, Ni level was comparatively higher at stage IV in the patients. The average concentrations of Zn and Cu were found more or less comparable in the scalp hair at all stages of lymphoma patients; however, the mean levels of Cr and Fe were comparable at stage I and stage III of the patients as shown in Fig. 6. Although the comparative study related to the types/stages of cancer revealed some significant differences, but these variations may also be partially ascribed to the disparities in the age, habitat and nutritional status of the donors as there were limited number of samples for some sub-group of the patients. Therefore, futuristic studies with larger number of samples are recommended to verify the present findings.

The comparative evaluation demonstrated the deficiency of some essential elements particularly Zn and Fe and excess of toxic elements of Cd, Cr and Ni in the patients compared to the counterpart controls. Elevated concentrations of toxic elements and deficiency of essential elements in the patients indicate an imbalance which may be linked with variety of pathological conditions including lymphoma^23–25^. Iron is an essential nutrient that facilitates the processes of cell growth, cellular metabolism and proliferation. It has long been known that Fe metabolism is altered in the presence of inflammation or neoplasia. Malignant cells demonstrate a greatly increased requirement for Fe as a result of their rapid cell division, which in turn altered iron homeostasis in cancer cells^26^. Due to the enhanced dependence on Fe metabolism in cancer cells, Fe depletion might have an anti-proliferative effect on tumour cells^27^. Moreover, Fe depletion causes a shift in cellular metabolism from oxidative phosphorylation to glycolysis, reducing the total number of ATP molecules available for cellular processes. Several epidemiological studies indicated that anaemia was a major clinical problem for lymphoma patients and was present in approximately 40% of HL patients^28^. Iron concentration was found lower significantly in the blood but was elevated in the hair of lymphoma patients compared to the healthy subjects^15^. In a recent study, Fe showed low concentration in the hair of the lymphoma patients than the normal subjects^12^. Furthermore, concentration of Fe was lower in dogs with lymphoma compared to the normal dogs^29^.

Zinc is a well known nutritionally essential element for humans, which prevents the formation of free radicals and is an important constituent of RBCs, transcription protein p53 and other DNA repair proteins^30^. It also increases circulation of T-lymphocytes in the blood and also enhances the ability to fight infection. Multiple studies have revealed that Zn deficiency causes T-cell dysfunction, impair cellular immune functions, dysfunction of neutrophils and natural killer cells^13^. Alternatively, Zn deficiency and its associated immune suppression may represent a paraneoplastic syndrome secondary to certain types of cancer^31^. More recently, some studies reported that Zn is also involved in homeostatic regulation and its deficiency, through increased oxidative stress accelerates the carcinogenic process^32^. Gupta *et al*.^33^ reported low concentration of Zn in malignant lymphoma patients. Cavdar *et al*.^34^ reported low Zn concentration in Turkish children with Hodgkin’s disease. It was observed that chronic Zn deficiency was linked to malignant lymphoma in Turkish children prior to any treatment^13^. When Hodgkin disease patients were compared to the controls, Zn concentration was significantly lower as examined by Cunzhi *et al*.^35^. In another report, it was found that mean Zn concentrations in the hair and blood of lymphoma patients was lower than the controls^15^. In a recent study, Zn level was exhibited lower in lymphoma patients than the controls^12^. Similar findings have been observed in the present study (Table 3). It has demonstrated that the development of lymphoid organs was seriously inhibited by the deficiency of Zn in chickens which further impaired the progression of lymphocytes and caused pathological injury in the lymphoid organs^36^. Dogs with lymphoma have been found to possess lower Zn concentration compared to normal dogs, however the cause of decreased Zn level was unknown^29^. However, in animal model, Zn deficiency was observed in all the three types of malignancies studies including spontaneous, induced and transplanted lymphomas when compared to their normal counterparts^37^.

Recent studies have shown that carcinogenicity due to some metals (Cd, Pb, As, Cr, Ni and Hg) is the result of the reactive oxygen species (ROS), which consecutively produces oxidative stress and cytotoxicity in the biological systems^20^. Oxidative stress can intensify lipid peroxidation and causes DNA fragmentation, protein oxidation and the reduction of enzyme activity. Several lines of evidence have suggested that toxic elements are closely involved in the initiation processes of cancer such as lymphoma^20,38^. Cadmium produces oxidative stress and it induces increase in ROS formation, which could indirectly result the disturbance of the cellular redox balance in favour of the pro-oxidants and can lead to disruption of cellular macromolecules^39,40^. There are many studies reporting a role of Cd in lung, renal, liver, breast, haematopoietic system, bladder, stomach, prostate and pancreatic cancers in humans^40,41^. Kelly and his colleagues indicated an association between Cd and NHL in females; however no such association was found in males^24^. Nonetheless, an association between urinary Cd exposure and NHL in males was observed^42^. In another study, Czerny and his colleagues observed increased concentration of Cd in lymphoma patients compared to the controls^12^. In the present investigation, it was found that Cd contents in cancer patients were appreciably elevated than the counterpart controls, indicating a critical role in the development of disease (Table 3). Cadmium induced tumours of the haematopoietic systems in rats and mice as well. Oral Cd induced dose-related increase in the incidence of leukaemia in male Wistar rats. Increased in lymphoma induced by injections of Cd in several strains of mice was observed^43^.

Oxidative stress produced by Cr, leads to the formation of ROS, induced genotoxicity, cytotoxicity and apoptosis or cell death which plays a significant role in lymphoma carcinogenesis^44^. Malignant lymphoma including Hodgkin’s disease also occurred more frequently in workers with higher exposure to Cr compounds^45^. In an investigation, it was notable that Cr as well as other chemicals had direct carcinogenic effect on Hodgkin’s disease^7,46^. The incidence of different malignancies including lymphoma was increased in rats with metal implants containing Cr^47^. On contrary, dogs with lymphoma had lower Cr concentrations than normal dogs and it was unclear whether decreased in Cr concentration in dogs with lymphoma were clinically relevant^29^. Chromium and its compounds have been tested for mutagenicity in several number of genotoxicity assays, from bacteria to laboratory rodents and human cells. Animal and epidemiological studies have consistently found positive results in all standard mutagenicity tests, including lymphoma cells in humans^25^. In present study, Cr revealed markedly elevated levels in patients (Table 3). Although Cr (VI) is considered as human carcinogen, during the present investigation Cr was analyzed as total Cr and no speciation was carried out for Cr (VI) or Cr (III).

Nickel indirectly damages cellular material by facilitating production of free radicals generated by ROS, disrupting cellular redox status and sustaining lipid peroxidation. Oxidative stress has been found to play a key role in Ni-induced toxicity and carcinogenicity^4,38,48^. Nickel concentration in NHL patients was elevated in comparison with the control group^49^. The high concentration of Ni can be explained by the high metabolic activity of malignant tissue which leads to an increase in intracellular accumulation of metal ions. Freitas *et al*.^50^ reported that Ni can trigger oxidative burst in human neutrophils. In cultured human lymphocytes, Ni induces oxidative stress^48^. The ability of Ni to enhance apoptosis may disrupt the physiological activities of neutrophils, with potential impact in its immunological and antimicrobial role^50,51^. A study revealed that Ni concentration in the blood and hair of lymphoma patients was significantly higher as compared to the controls^15^. Significantly higher Ni levels in the blood and scalp hair of the patients compared to the controls was detected in the present study, clearly indicating the adverse effect of Ni overload in the patients (Table 3).

Lead induces DNA damage via elevated level of oxidative stress and is known as carcinogenic, nevertheless, epidemiological evidence in humans is less consistent and lacks a clear dose-response relationship. However, the evidences from animal models are strong; experimental studies suggested increased incidences of renal cancer and to a lesser extent brain, lung and haematopoietic cancers, among rodents exposed to lead^24,52^. The results of a study conducted in Peshawar, Pakistan revealed that the mean Pb concentration increased significantly in lymphoma patients than the controls^15^. In the present investigation, Pb levels were noted relatively higher in the controls compared to the patients (Table 3).

As a vital component of superoxide dismutase (SOD), Mn has important antioxidant properties since MnSOD plays a significant role in protecting cells from oxidative damage by ROS and tumour suppressor. This MnSOD reported deficient in lung and breast cancers^53^. Moreover, Mn is needed for colon cancer cells to bind to extracellular matrix proteins during migration and metastasis^54^. Clearly, the relationship of Mn to cancer is complex, and data are somewhat conflicting about this metal’s role. Moreover, Agency for Toxic Substances and Disease Registry found no evidence of cancer effects in humans exposed to Mn^55^. However, exposure to excessive Mn level induces apoptosis in cultured cerebellar granular neurons cells^56^. Various lines of evidence suggested that Mn induced oxidative stress and play an important role in diffuse large B-cell lymphoma^57^. At cell level, Mn preferentially accumulates in mitochondria, where it disrupts oxidative phosphorylation and increases the generation of ROS^58^. Results derived from the present work revealed that the Mn levels were decreased in the patients versus controls (Table 3).

Copper is an essential element and an integral component of many metalloenzymes, but excess of Cu has been considered as potent oxidant, causing the generation of ROS, are hallmarks of range of cancers, including lymphoma^30,59^. In human plasma, ceruloplasmin (Cu-carrying glucoprotein) levels are found to be elevated in breast cancer, lymphoma and gastrointestinal tract cancer^60^. Furthermore, it has been suggested that Cu concentration in the organism can be used as an index for assessing the extent of malignant lymphoma and might be a predictor for chemotherapy response^61^. Few studies have investigated the relationship between Cu and lymphoma and these studies were conducted in the past, almost 20 or even, 40 years ago^14^. Shah-Reddy *et al*.^62^ and Cohen *et al*.^63^ observed a positive correlation between Cu level and NHL. It was proven that measuring Cu level could be useful in assessing disease activity and response to treatment in some kinds of lymphoma and has been found in some literature to be a diagnostic factor^64^. Cohen *et al*.^63^ and Wu *et al*.^65^ have reported a significant difference in Cu of NHL patients with advanced disease patients (stages II–IV) compared to healthy controls. Other authors suggested that all patients with low grade and high-grade NHL have elevated Cu level compared to the control group^33,49^. In another study, a significant difference was also found between Cu and copper/zinc of Hodgkin disease patients and controls and in the patients with different histopathological types of HL^35^. It was reported that Cu concentration in blood and hair was significantly high in lymphoma patients compared to their healthy relatives^15^. A case-control study in Canada found increased NHL risk among women living with proximity to Cu smelters^4,66^. Kaiafa *et al*.^14^ found an increase in Cu concentration in haematological malignancies than normal. However, Jamakovic and Baljic^23^ found no statistically significant correlation between mean level of Cu and HL. In the present investigation, Cu levels were noticeably elevated in the case of patients; it pointed out toxic contribution towards the progression of lymphoma (Table 3).

In the present study, we observed altered levels of the essential and toxic elements and believed that determination of elements in the blood and scalp hair might be useful for assessing the presence of lymphoma. Such alteration may prove to be useful markers to screen for and perhaps monitor relapse of malignant disease. The analysis of essential and toxic elements in biological samples of lymphoma patients and related healthy donors provides knowledge to oncologist about the importance of alteration in metabolism of the elements. This information may be used for the diagnostic and therapeutic purposes as well. However, as prognostic factors they did not seem very sensitive. Interestingly, some of the elements (such as Cr and Cu) which are essential for growth and development are also implicated in the genesis of cancer at sufficient exposure and in certain forms. Nevertheless, the study is not free of limitations. The findings of the present study could not be generalized to all the lymphoma patients because of the regional and cultural variation that occurs within and between the countries. Furthermore, in the present study the selected elemental levels were observed in relation to clinical staging of lymphoma, nonetheless due to small/limited number of samples, it was not possible to examine the clear associations among different histological types and stages of lymphoma patients possibly induced by the toxic elements. It has been revealed that the socioeconomic factors also play a role in higher mortality rates in the patients, such as poor nutritional environment and unequal access to health care due to poverty as the cost of treatment for lymphoma is very high. In Pakistan, shortage of the analytical data is a major limitation to draw sound conclusions. Inadequate and insufficient diagnostic procedures, lack of expert consultation, poor data repositories, uncertainty about the patients’ origin and food habits, as well as delays in the treatment are the major constraints for cancer registration in Pakistan. It is important to note that population-based surveys should be able to identify knowledge deficits about diagnostic procedures and risk factors in connection with specific socioeconomic positions. Therefore, occupationally and educationally, awareness of the frightful effects of these elements on lymphoma risk factors should also be emphasized. A far further study is warranted to more clearly define the risks associated with specific exposures with different specific histological types and grades of lymphoma having large number of subjects. It is also concluded that regular monitoring of toxic elements in biological/clinical specimen plays an important role in identifying possible sources of intoxication and contamination, as well as in preventing, through early detection, the onset of toxic elements related diseases including lymphoma.

## Conclusion

In the current study, measured concentrations of Cd, Cr, Cu and Ni were found significantly elevated in the blood and scalp hair of lymphoma patients than the controls. Furthermore, levels of other trace elements, such as of Zn (noticeably) and Pb & Mn (significantly) were found lower in the lymphoma patients than the controls, thus showing alteration of these elements between patients and controls. Some noteworthy variations were also exhibited in the blood and scalp hair elemental levels based on on cancer types, subtypes and stages, which are possibly related to the cancer aetiology or growth stage in patients. In NHL patients, Mn and Ni showed elevated levels in the blood while Pb, Fe and Ni levels were highest in the scalp hair. In HL, Cd, Pb and Fe levels were highest in the blood and Cr level was maximum in the scalp hair of patients. Average concentrations of Cu and Cr were highest at stage-I, Pb showed highest level at stage-III and Mn was elevated at stage IV in the blood of the patients. Similarly, average levels of Cr, Fe, Mn and Cd were highest at stage II, while Ni was elevated at stage IV in the scalp hair of patients. Correlation study exhibited noticeably different mutual variations of the elements in the blood and scalp hair of the two groups. PCA and CA revealed diverse apportionment of the elements in the blood and scalp hair of the patients and controls; thereby indicating that the carcinogenic processes were significantly affecting the toxic and essential elements balance in humans. Accordingly, statistical mode of analysis may be used as an additional tool for the prediction/advancement of the lymphoma in the patients, although more detailed studies with larger population segments should be conducted to authorize its inferences.
